# From Understanding the Development Landscape of the Canonical Fate-Switch Pair to Constructing a Dynamic Landscape for Two-Step Neural Differentiation

**DOI:** 10.1371/journal.pone.0049271

**Published:** 2012-12-04

**Authors:** Xiaojie Qiu, Shanshan Ding, Tieliu Shi

**Affiliations:** 1 Center for Bioinformatics and Computational Biology, and The Institute of Biomedical Sciences, School of Life Sciences, East China Normal University, Shanghai, China; Centro Cardiologico Monzino, Italy

## Abstract

Recent progress in stem cell biology, notably cell fate conversion, calls for novel theoretical understanding for cell differentiation. The existing qualitative concept of Waddington’s “epigenetic landscape” has attracted particular attention because it captures subsequent fate decision points, thus manifesting the hierarchical (“tree-like”) nature of cell fate diversification. Here, we generalized a recent work and explored such a developmental landscape for a two-gene fate decision circuit by integrating the underlying probability landscapes with different parameters (corresponding to distinct developmental stages). The change of entropy production rate along the parameter changes indicates which parameter changes can represent a normal developmental process while other parameters’ change can not. The transdifferentiation paths over the landscape under certain conditions reveal the possibility of a direct and reversible phenotypic conversion. As the intensity of noise increases, we found that the landscape becomes flatter and the dominant paths more straight, implying the importance of biological noise processing mechanism in development and reprogramming. We further extended the landscape of the one-step fate decision to that for two-step decisions in central nervous system (CNS) differentiation. A minimal network and dynamic model for CNS differentiation was firstly constructed where two three-gene motifs are coupled. We then implemented the SDEs (Stochastic Differentiation Equations) simulation for the validity of the network and model. By integrating the two landscapes for the two switch gene pairs, we constructed the two-step development landscape for CNS differentiation. Our work provides new insights into cellular differentiation and important clues for better reprogramming strategies.

## Introduction

The canonical view of differentiation as an irreversible process has been largely reshaped since the emergence of induced Pluripotent Stem Cells (iPSCs) and other lineage conversions techniques in stem cell biology [1]–[8]. The success in inducing a conversion between cellular fates raises several questions [9]: why is a stable mature cell type retrodifferentiable or convertible? Is there a universal principle that can explain cellular development, and is there a fundamental commonality shared by the processes of normal differentiaton, retrodifferentiation and transdifferentiation? What are then the differences among the three processes?

In fact, a first effort to find a general principle traces back to Waddington’s pioneering work in embryogenesis which gave rise to his “epigenetic landscape metaphor” (Fig. S1) for development [10]. Here the landscape metaphor describes differentiation as a “down-hill” process, which is about a cell “rolling” down from the pluripotent hilltop (the embryonic stem cells) to the lower valleys (the terminal differentiated cells), with multiple bifurcations at the watersheds on the landscape [10]. This metaphor, apparently lacking physical basis in Waddington’s time, has long been ignored by experimental biologists until seen a renaissance among them in the recent years [11],[12]. Theorists had revisited this problem at various times. About twenty years after the first revelation of Waddington landscape, Thom proposed the catastrophe theory to explain the branching process in biological system [13]. However, he failed to find a potential function to construct the landscape. Kauffman, in a perspective different from that of Thom, starting from the idea of complex gene regulatory networks (GRNs) proposed that cell types are attractors in GRNs [14],[15]. His work used an efficient mathematical tool - Random Boolen Network (RBN). In parallel, more detailed modeling approaches (like Ordinary Differential Equations or ODEs) were also increasingly applied in modeling gene regulatory circuits. However, detailed studies of differentiation remain scanty and theories from dynamical systems have been applied only recently to the analysis of gene regulatory networks in development, starting with a single binary cell fate branching process [16]. Subsequently, the proposal of “sequential branching” model for hierarchical determination of cell fates, implemented both as ODEs or Stochastic Differentiation Equations (SDEs), has led to insights of how gene network dynamics govern pancreas development [17]. The re-discovery of intrinsic stochasticity [18] of gene expression in mammalian cells as well as developments in the theory of stochastic process has led to a first formalization attempt of the “arrow of time” (time-directionality) for cellular differentiation [19]. In parallel, several studies on constructing the potential landscape for different biological systems now finally begin to address Thom’s problem [20],[21].

In nonequilibrium systems, an explicit potential function generally does not exist, and an intuitive solution is to relate some form of potential to the steady state probability distribution in stochastic system and decompose the force driving the system into the gradient part and the curl part. Using such an approach, Wang constructed the landscape for the cell cycle dynamics and proposed that the curl force is responsible for the dynamics of a limit cycle [22]. Of crucial importance, these theoretical developments led to the first formalization for the one-step of binary branching in Waddington developmental landscape, generating myriad implications in explaining development and reprogramming [23]. However, in these models dimension of the developmental process on the landscape is represented by a hypothetical change of a specific model parameter whose physical validity is not confirmed. Therefore, it is necessary to study the meaning of this operation. In addition to Wang’s method, there exists other constructive and *ad hoc* methods for landscape construction [24]. Even before Wang’s construction, Ao proposed a transformation of the SDEs to obtain a potential function for constructing the landscape, similar to finding an effective flux in the Helmholtz-Hodge decomposition [25]–[29]. Based on the intuition from Lyapunov theory, Bhattacharya developed a numerical framework for mapping the quasi-potential landscape of a two-gene system [24]. However, none of them has been used to construct the landscape based on a real gene circuits governing the development across two steps of cellular fate branching.

Here we first quantified the dynamic landscapes for the canonical two-gene fate switch motif under different parameter changes and demonstrated that some change can correspond to a development process with its monotonic increase of entropy production rate (EPR). We then extended previous work to quantify the transdifferentiation paths in addition to the previously quantified differentiation and retrodifferentiation paths on the landscape by using the theory of least action path. We further applied our theory to cell differentiation in the central nervous system (CNS) as an example to construct the two-step developmental landscape. The CNS was chosen because of the extensive researches about neuronal cell differentiation, which have accumulated abundant experimental data on gene regulatory circuit suited for modeling [30]. Intensive theoretical efforts in developmental neurobiology also have persisted for the past thirty years [31]. A considerable proportion of those work have concentrated on signaling cascade and morphogenesis in the neural development. However, GRN has dominant influence when it comes to the differentiation from nervous precursor to three CNS cell types (neuron, astrocyte and oligodendrocyte). Thus here we mainly model gene expression dynamics underlying CNS differentiation, first through qualitative assessment of a core regulatory network, followed by comparing the stochastic simulation (which was also used to explore the optimal cocktails for reprogramming from astrocyte or oligodendrocyte to neuron) with microarray data to confirm the model and network’s validity. By gluing the landscape for two fate-switch motifs, we finally constructed the two-step landscape which has never been done before to our knowledge. Our work provides new insights to general principles about the mechanisms of retrodifferentiation and transdifferentiation.

## Materials and Methods

### Two-gene Landscape

**Figure 1:**
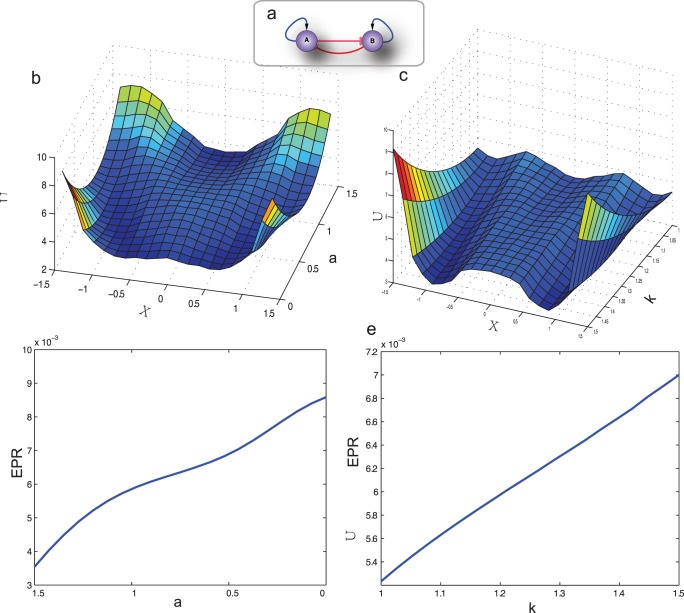
The two-gene developmental landscapes and entropy production rate (EPR) evolution under parameter *a* or *k* change. (a)Two-gene model of cellular development with self-activation and mutual-inhibition. The results illustrated in following rows are based on this model. **First row**: The landscape can be equally constructed by changing different parameters in Eq. 1. *X*-coordinate corresponds to the gene expression of *x*_1_ and *x*_2_ after a coordinate-transformation (see main text) while the ordinate coordinate corresponds to the parameter change, which is used to model the process of development. The *U*-coordinate describes the quasi-potential. The center valley on the landscape corresponds to stem cell type while the two side valleys correspond to two different terminal cell types. (b) *a*-change landscape constructed by change of parameter *a* with other parameters fixed. The same treatment is performed for the subgraph c. (c) *k*-change landscape **Second row**: The EPR increases monotonically along the change of parameter *a* or *k*, supporting the idea that parameter change during landscape construction can correspond to a self-organization thus the development process. (d) EPR vs. *a* decreases from 1.5 to 0. (e) EPR vs. *k* increases from 1 to 1.5. Basic parameters are set as 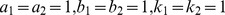 and 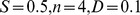.

Recently a large range of cellular fate-switch regulator pairs, characterized by their cross-inhibition and self-activation, are confirmed [32]. This common gene pair is increasingly regarded as a general network design for the cellular fate bifurcation [33] (Fig. 1a). In order to introduce the developmental landscape idea and demonstrate how a landscape can formulate the global developmental (also the reprogramming) processes mathematically and physically, we studied the canonical two-gene module (Fig. 1a) as our first step. To characterize dynamics of this two-gene network, we assumed that autocatalysis and mutual-inhibition of genes are independent (their effects are thus additive), and thus used the following standard kinetic model [16],[23]:
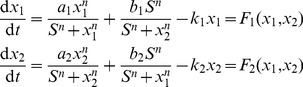
(1)in each of the above two nonlinear equations the first term represents the contribution to rate of change in gene expression from autocatalysis where the transcription and translation processes are lumped into one step; the second term represents that of inhibition from another gene and the last term first-order degradation. The first and second terms are implemented in the form of Hill equation with *n* = 4, but do not necessarily imply the cooperation between regulators [34]. Basically, we set 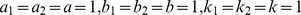, and *S* = 0.5 to ensure simplicity and symmetry of the model. The rate of change in gene expression can also be regarded as a force driving the gene network. In vector form, we obtain:
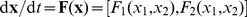
(2)


In order to obtain a global and intuitive understanding of the development process described by the above kinetic equations of two fate-switch genes, a developmental landscape under the concept of Waddington landscape is needed [23],[35]. Until recently, there are three different approaches available for landscape construction [23],[24],[26]. In the following we derived the formula for landscape construction from Wang’s framework [23], which relates the quasi-potential to the steady state probability of a biological stochastic system. The mathematical basis of two other methods [24],[26] and the corresponding two-gene landscapes are included in the supporting information (Fig. S2).

In nonequilibrium system with 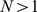 dimensions, it is not easy to obtain a potential function *U* which satisfies 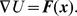 Since the intrinsic and extrinsic noise exists universally in biological system, an intuitive solution is to relate some form of potential (quasi-potential) to the steady state probability distribution. The above equations under noise influence can be related to a continuous stochastic model, whose probability distribution evolution is determined by the diffusion equation.

According to the probability conservation law (The probability (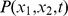) change at point (*x*_1_,*x*_2_) equates to the sum of the flux 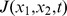 flows in/out of that point), we obtain the following equation:
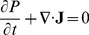
(3)where probability flux is defined as 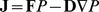 based on the following Fokker-Planck (FP) equations:
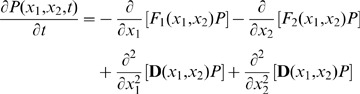
(4)where D is the diffusion matrix which is set as a constant (which is basically 0.1) in our simulation for simplification.

In steady state, the 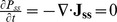 but probability flux 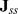 itself doesn’t need to be zero, therefore,
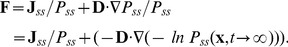
(5)


Following the Boltzmann law in equilibrium system, the steady state probability can be converted into the dimensionless quasi-potential according to:
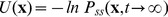
(6)Then,



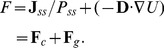
(7)The force driving the nonequilibrium system is thus decomposed into two parts: the gradient term 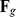 (relates to the steady state probability) and the curl term 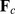 (relates to the divergence-free flux).

Even though the potential in nonequilibrium or equilibrium system relates to steady state probability distribution in a similar way, the dynamics of the nonequilibrium system is also determined by the curl force 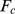 in addition to the gradient force 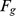. The obtained quasi-potential is then used to map stage-specific and whole dynamic landscape (Fig. 1b, c & Fig. S3A, B).

**Figure 2:**
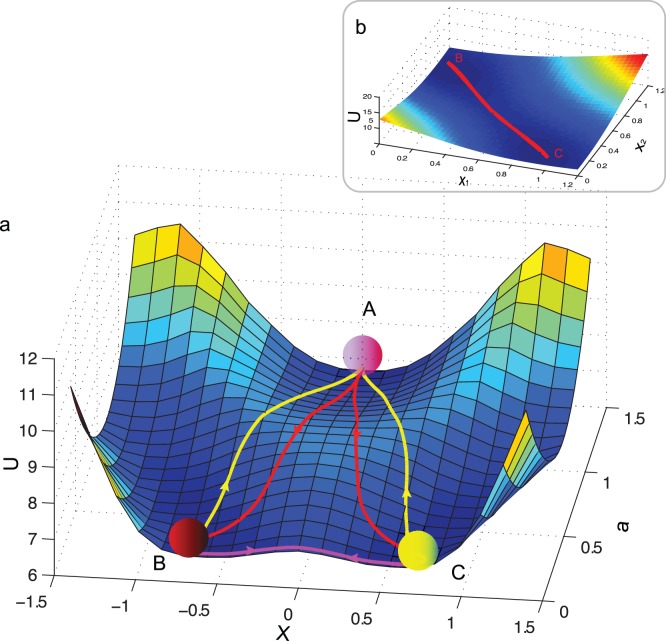
The development, retrodifferentiation and transdifferentiation paths over the landscape. As indicated in the landscape, the retrodifferentiation path is distinct from the developmental path. Transdifferentiation path (while 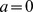) has an intriguing behavior, which directly traverses from the source valley to the target valley without change of parameter *a*. The corresponding dominant transdifferentiation paths on the stage-specific landscape are nearly identical (Shown in the inset b). The arrow indicates the direction of the dominant path. A, B, C are symbols for stem cells and two different terminal cell, respectively. (a) Development (from A to B/C, red color lines), retrodifferentiation (from B/C to A, yellow color lines) and transdifferentiation (between B and C, magenta color lines) paths over *a*-change development landscape. (b) The forward and backward transdifferentiation paths on the stage-specific landscape at 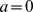.

Following Wang’s method, the change of parameters is used to represent the dynamic process. By fixing parameters 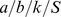 for 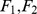, we calculated *U*, the quasi-potential for each point in the gene expression state space of 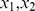 from the above equation, and then constructed the stage-specific landscape, which is defined because the parameters in the model are constants thus we can relate the landscape to a dynamic stage. The stage-specific landscape was further manipulated to construct the whole dynamic landscape. We rotated anticlockwise the 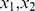 coordinates and the landscape 45°, followed by extracting the lowest *U* for each value at the new coordinate *X* (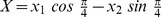) which was then used to represent the potential for a specific stage. We then systematically changed one of four parameters to represent the whole dynamic process and quantified the corresponding stage-specific landscape. After combining a serial of that set of potential, we mapped out the whole dynamic landscape. The EPR (Entropy Production Rate) is used to explore the relationship between change of 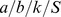 and the development process (Fig. 2d, e & Fig. S3C, D). An important characteristic of a self-organization system (including the living system) is that it subjects to the principle of maximum entropy production rate (MEPR), that is the EPR will increase as the system evolutes into more ordered states (corresponding to the process that cells develop into specific mature cell types) [36]. Therefore, we assumed that the monotonically increase of EPR for parameter change in the landscape construction can be related to a developmental process. The formula of EPR is defined as [37]:
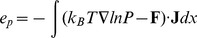
(8)where 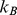 is the Boltzman constant. In the dimensionless condition, we can use 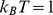. For the dynamic landscape where the EPR increases monotonically along the change of parameters on the landscape construction, they are regarded as the “verified development landscapes”.

Different cell types can be associated with different valleys (attractors) on the landscape [14]. Under the principle of least action, the dominant paths between the stem cell type valley and terminal cell type valleys can be quantified [23]. These dominant paths are regarded as the biological development (paths from stem cell valley to two terminal cell valleys), retrodifferentiation (paths from terminal cell valleys to stem cell valley) and transdifferentiation (paths between two terminal cell valleys) processes (Fig. 2). The dominant paths were obtained by minimizing the following action of the paths, for which we used the simulated annealing algorithm [23],[38].
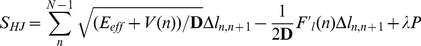
(9)


The theoretical details about how to derive the above equation can be referred to Wang’s papers [23],[38]. To find the transdifferentiation paths, two searching methods were used. The first one was operated under the symmetrical change of parameter 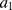 and 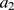, while the other was operated under asymmetrical change of parameter 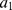 and 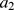, which means that we fixed 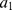 and changed 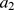 if we wanted to transdifferentiate the cell from 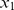 high expressed cell type to 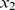 high expressed cell type, or *vice versa*.

**Figure 3:**
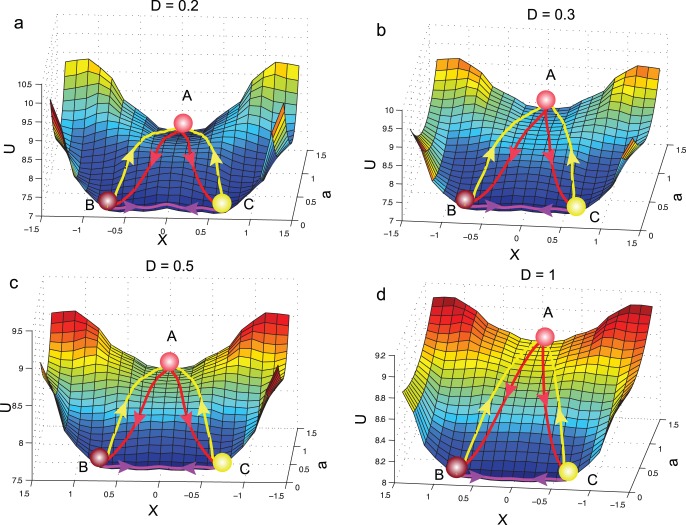
The developmental landscape and dominant paths under different noises. As the noise increases, the developmental landscape becomes flatter while the dominant paths on the landscape become increasingly straight. Thus the cell types become less stable and the development (and also the retrodifferentiation and transdifferentiation) process(es) becomes more flexible. The same conclusions apply to *k*-change landscape (Here it is the *a*-change landscape). This graph demonstrates the importance of stochasticity in cellular development. (A–D) Landscape and dominant paths when 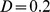, 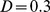, 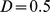 and 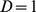, respectively. Red color lines: development paths; yellow color lines: retrodifferentiation paths; magenta color lines: transdifferentiation paths.

We further studied the effects of noise over the landscape topography and the dominant paths by varying the noise ratio from 0.1 to 1 (Fig. 3).

### Minimal Network Responsible for CNS Differentiation

The commitment of neuron lineage depends on the lateral inhibition, in which the prefated neural progenitors inhibit the neurogenesis of the neighboring cells through the 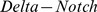 pathway. Proneural genes, like 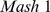, in the downstream of this pathway, are crucial for neural induction [39]. Their inhibitors, such as 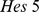, on the other hand, are glia inducers [39]. However, the key regulators which are in charge of the astrocyte-oligodendrocyte fate switch have not been fully identified, excepting limited reports like decreasing of 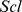 leads to 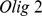 high expression, and results in the formation of oligodendrocyte. For the purpose of modeling, we need to construct a minimal network for CNS differentiation by integration of scattered evidences available. We focus on the master genes, and do not consider the signaling molecules and influences from cellular environment in our model, as stated in the section of introduction. By doing so, we collected genes responsible for CNS fate decision and lineage markers genes. In particular, we started the network construction from finding 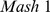, 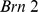, 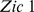, 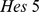 and 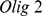
’s upstream regulators or targets because they are the five factors used in the lineage conversion from fibroblast to functional neurons, implying their importance in the CNS differentiation [5].

**Figure 4:**
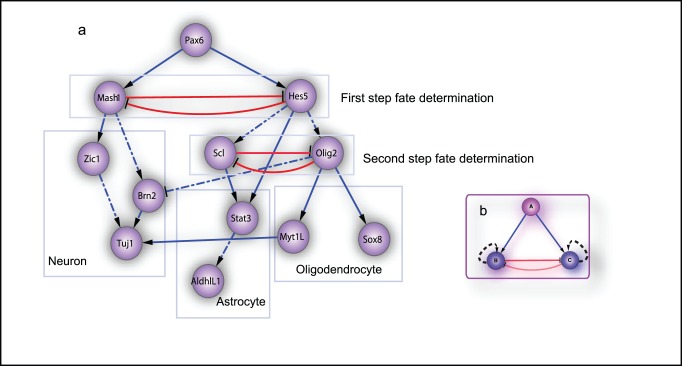
Manually-curated minimal GRN for central nervous system differentiation and network design explaining differentiation. (a) The manually-curated minimal network for CNS differentiation. The network mainly consists of two steps of two mutually-inhibited fate commitment genes (boxes with red color lines): the first step determines the commitment of neurons or glia where the second step determines that of astrocyte and oligodendrocyte. From left to right, the three boxes below the fate switch circuits includes neurogenesis, astrogenesis and oligodendrogenesis related transcription factors and markers, respectively. Real lines are literature confirmed regulations while dotted lines are proposed regulation in CNS development (See Tab. S1). The skeleton of this network includes two coupled three-gene networks in subgraph b. (b) The basic motif for the CNS differentiation network where one progenitor gene (A) activates the expression of two mutual-inhibited downstream genes (B and C). This motif (with the self-activation) also corresponds to our proposed network design for explaining differentiation where gene A represents OKSM while B and C two distinct fate commitment genes.

The curated minimal network responsible for the differentiation of CNS is shown in Fig. 4a which is characterized by the coupling of two-step fate decision. References supporting this network are summarized in Tab. S1.

### Model the GRN Dynamics of CNS Differentiation

We model the network in Fig. 4a as previous work [17]. The formula of gene expression change rate in the SDEs (Eq. 11) for the neural differentiation network contains three terms. The first term describes the gene’s interaction with other genes (or itself), which is implemented in the form of a customary Hill equation. To reduce the computational complexity for high dimension system while focus on the qualitative behavior, we minimize the number of rate constants and combine the activation and repression effects into a single term. The expression of positive regulator will appear in both of the denominator and numerator of the regulated gene’s equation; while for a negative regulator, it will appears only in the denominator. We multiply their expression to model the cooperative regulations between different genes. 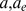 are used to represent different levels of production rate. The second term of the equations reflects first order degradation. To capture the gene expression stochasticity, we also added a Gaussian white noise as the third term. The noise is defined as elsewhere,
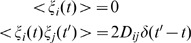
(10)where the first equation means that the ensemble average of the noise is zero and the second equation describes the independence of the noises between different time points, implying a Wiener process. The set of SDEs for the twelve genes in Fig. 4a is defined as:



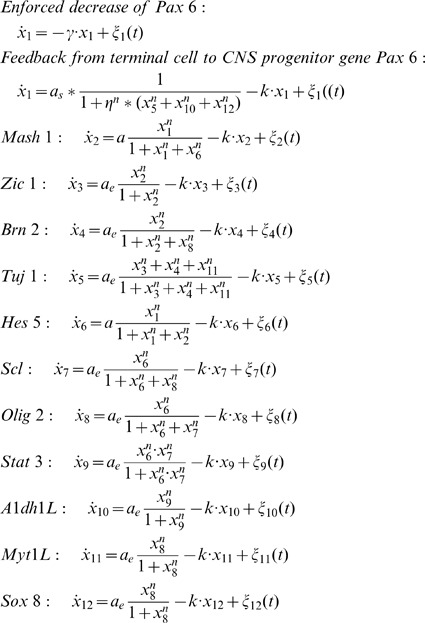
(11)The parameters of the above equations in our simulation are set as: 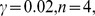 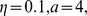 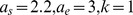. Several other sets of parameters were implemented and similar qualitative results were obtained. To represent the negative feedback of the mature terminal cells back to the precursor gene 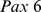, for which there is functional but no molecular evidence, we enforced an exponential decrease of 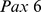 in our model (the first equation for the change of 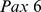). However, we obtained qualitatively similar conclusion when the differentiation process was modeled by negative feedback from terminal cells to the 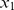 (the second equation for the change of 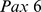, Fig. S5A, B). The equations for the two three-gene motifs (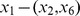 and 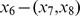
in Fig. 4a) are the core of the CNS development system, which was further used to constructed the CNS developmental landscape.

#### Verification of the model by SDEs simulation

**Figure 5:**
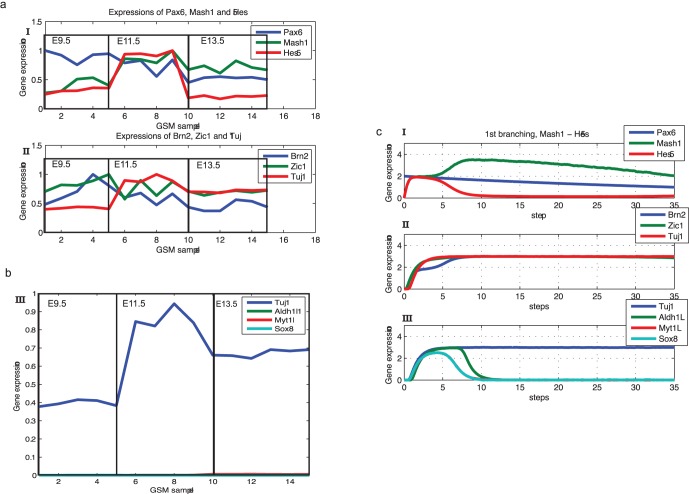
Results of stochastic simulation matches qualitatively public microarray datasets for neurogenesis. Regulators for neurogenesis and marker genes of three cell types were chosen to compare the result of stochastic simulation and microarray data. (a) Microarray data for regulators and markers of neurogenesis from 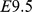 to 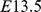. Each box represents a specific time period, in which there are five duplications of samples. Diagrams in row I and II shows the gene expression dynamics for differentiation inducer and the first-step switch genes, and neural related regulators/markers, respectively. The expression level of each gene is normalized by the corresponding maxima in the time series. (b) Relative expression dynamics of three cell types marker genes in neurogenesis by microarray data. Here the expression 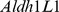, 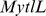 and 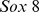 is much lower than that of 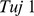, thus is shown near the horizonal axis. This diagram corresponds to the low expression of 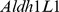, 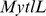 and 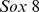 in the late part row 3 in subfigure c. (c) Single cell simulation for neurogenesis where diagrams in row I and II matches quantitatively with the gene expression dynamics of the diagrams in the according rows of subgraph a.

After searching the GEO [40], three datasets were retrieved as the reference for the stochastic simulation (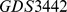, 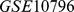, 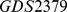 [41]–[43], which study neurogenesis, astrogenesis and oligodendrogenesis, respectively). For each dataset, we first normalized the expression levels of specifically chosen genes by their maxima in the time series. By doing so, we can quantitatively demonstrate the expression pattern during neurogenesis which can be used to compare the simulated gene expression dynamics (Fig. 5a & Fig. S6–7A). We also compared gene expression level of marker genes during three different CNS cell type genesis (Fig. 5b & Fig. S6–7B). We made single cell simulation (one run of simulation) with Eq. 10 and compared the simulation dynamics with that of the corresponding genes from microarray datasets (Fig. 5c, Fig. S5 & Fig. S6–7C).

### Central Nervous System Developmental Landscape

It is impossible to visualize a landscape of minimal CNS differentiation network which has twelve genes. Therefore we made a reasonable simplification: we mapped the landscape based on gluing two binary fate-decision modules’ landscapes since they are the master regulatory pairs responsible for CNS cell types’ fate commitment and the downstream genes don’t have impact on the upstream genes due to the fact that we ignored the feedback from the downstream genes in our simplified model. Each fate-switch landscape was constructed for the corresponding three-gene module in a similar way as the previous two-gene developmental landscape construction (the remaining nine dimensions were used as the parameters of the module’s equations).

**Figure 6:**
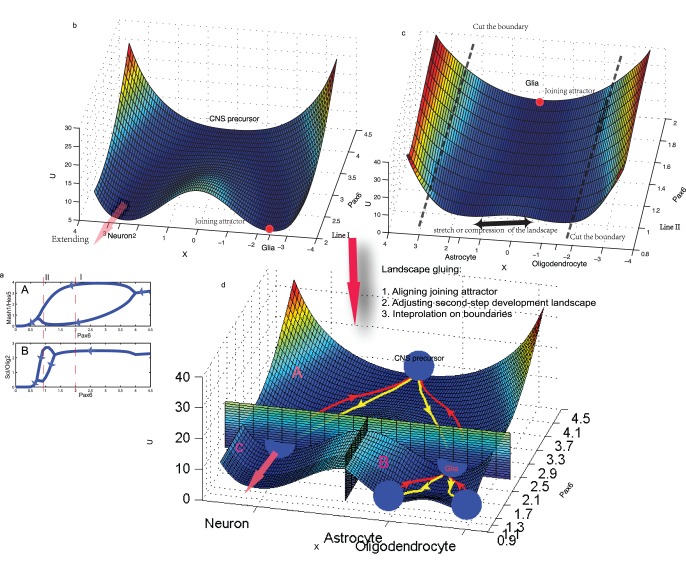
The two-step CNS developmental landscape. To map the two-step landscape for CNS differentiation, we first calculated the bifurcation diagram of fate-switch genes using the gene expression of 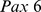 to represent the development process. Based on this bifurcation diagram, we identified the parameter region for the first and the second step development. Then we calculated and glued the first-step and the second-step landscape in an *ad hoc* coordinate system to obtain the two-step developmental landscape, which is similar to the original Waddington’s landscape. (a) The bifurcation diagram for two fate-switch genes motifs under the change of 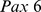. Here we only showed the stable fixed point. Line I indicates the boundary for the first-step differentiation, which is used in constructing subfigure b (the first-step landscape). Line II indicates the boundary for the second-step differentiation, which is used in constructing subfigure c. The cut-off values of 2.0 and 0.9 at the line I and line II are the values where the bifurcation reaches the largest region and that the corresponding differentiation step is assumed to be completed. (b) The first-step landscape for 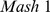-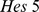 motif which describes the CNS precursors develops into neurons and glia. The left neuron valley of the landscape will be extended during the construction of CNS development landscape. (c) The second-step landscape for 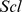-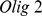 motif which describes glia develops into astrocyte and oligodendrocyte. The mesh size in mapping this landscape can be adjusted to align the joining attractors from first and second-step landscapes. This landscape was further compressed and its left and right boundaries were cut off during following construction of the CNS development landscape. (d) The two-step CNS developmental landscape. This landscape is constructed by gluing the first and the second step development landscape and the extending landscape, respectively symbolized as region A, region B and region C. Three specific steps were involved during the gluing of the CNS development landscape. The dominant paths are also shown on the landscape. Yellow color lines represent the development processes while red color lines represent the retrodifferentiation processes. The transdifferentiation paths need firstly move back to the CNS progenitors and then downward to the target cell types (Data not shown). Thus the transdifferentiation paths can be regarded as a combination of retrodifferentiation and dedifferentaition processes.

In constructing the whole landscape, the expression level of 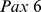 (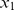) was used to represent the development stage (Fig. 6a), consequently it can be regarded as the bifurcation parameter of binary fate commitment. We first visualized the bifurcation diagram for the motifs of 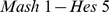 and 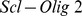 and identified the ranges of bifurcation parameter (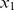) for the first and the second bifurcations (Fig. 6a). The range of expression level of 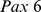 for the first bifurcation is about from 4.5 to 2.0 while the second bifurcation is about from 2.0 to 0.9. The cut-off values of 2.0 and 0.9 are the values where the first and the second bifurcations reach the most remarkable region (Line I and II on Fig. 6a), respectively. Therefore, we assumed that at 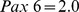 the neuron matures and glia forms from which the second-step differentiation starts while at 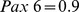 the astrocyte and oligodendrocyte mature and the second-step differentiation stop. In order to construct the two-step CNS developmental landscape, we then mapped the first and the second-step switch genes’ landscape according to the parameter ranges of bifurcation. Specifically, we used the decrease of expression level, from 4.5 to 2.0, of 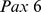 or 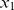 (similar to the parameter *a* in two-gene landscape) to represent the first step differentiation, then calculated the quasi-potential of 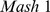 and 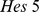 state space at different 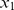 by solving corresponding FP equation and finally mapped the first-step developmental landscape (Fig. 6b). Similarly, we used the range from 2.0 to 0.9 of 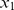 to represent the second-step differentiation. We then found the attractor states of 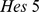 high expression at each 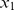 value and substituted the expression value of 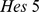 at each attractor into the equations of the second-step fate determinants (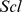 and 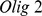) and finally mapped the second-step developmental landscape after solving the FP equation (Fig. 6c).

We used an *ad hoc* coordinate system to integrate the two landscapes into one. When the ordinate coordinate is between 4.5 and 2.0 (expression level of 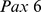), the *X* coordinate corresponds the transformed (similar to the two-gene landscape transformation) gene expression of 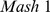 and 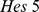 and the corresponding region (Region A in Fig. 6d) represents the first-step landscape. While the ordinate coordinate is between 2.0 and 0.9, the left part of *X* coordinate corresponds to the extending value of the previous coordinate while 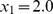 (Region B in Fig. 6d). The right part corresponds to the compressed 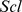 and 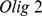 coordinate system (Region C in Fig. 6d, see explanation below).

In order to glue the first-step development landscape (region A in Fig. 6d), the extending neuronal landscape (region C) and second-step development landscapes (region B), three specific processes are involved. Firstly, the attractors on the joining of the two landscapes (while 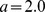) are aligned. In practice, the average height of the second-step landscape (including that of the joining attractor) can be adjusted through the size of mesh used in calculating the underlying probability with smaller size a larger average probability (*P*) per unit area and thus lower height (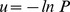) for the landscape (The probability used in calculating the landscape amounts to 1). By adjusting the size of mesh, we obtained a similar potential for the joining attractors. Secondly, while fixing the joining attractor, the second-step landscape can be stretched or compressed horizontally to maximally matching the boundaries near the two joining attractors since the *X*-coordinate is *ad hoc* and that of first and second-step doesn’t have direct relationship. Lastly, for the “bumps and cliff” remains between the joining of regions A, B and C, we first cut off parts of the boundary of the second-step landscape with high altitude and then used interpolation to create a smooth transition between different regions. This is warranted for that the boundaries with very high altitude have very low probability (which in reality is possibly of nonexistence) thus can be disregarded and kept as spaces for the interpolation. On the other hand, the barrier between different valleys obtained after interpolation is enough to describe the stability of cell types and the difficulty of transdifferentiation between neuron and astrocyte (or oligodendrocyte).

## Results

### Two-gene Developmental Landscape

We generalized Wang’s framework to further quantify the landscape under other parameter change and to explore whether or not the change can relate to a development process.

We found that, while fixing other parameters, varying of parameters: 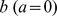 (the measure of inhibition), *k* (the measure of degradation strength), *S* (the reaction threshold constant), in addition to parameter *a* (the measure of self-activation strength) in Wang’s case [23], we can map similar landscapes (Fig. 1a, b and Fig. S3A,B). The *b*-change landscape is different from others in that the landscape (Fig. S3A) undergoes a bifurcation from one attractor to two attractors analogous to type *I* bifurcation in ref. [16].

Previously Wang’s work [23] demonstrated that the change of parameters leads to the bifurcation. Is this reasonable to represent the developmental process on landscape construction? Do they have certain physical relevance with the development process? We were thus motivated to explore the relationship between the change of parameters and the entropy production rate (EPR, Eq. 8). In Fig. 1d, e, we show that the EPR increases monotonically along the corresponding change of 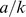 in constructing the landscape. Therefore, we demonstrate that the change of 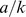 in the landscape construction may be applicable to characterize the developmental process. When parameter *b* increases from 0 to 1.5, however, the EPR does not always increase monotonically (Fig. S3C). The starting non-monotonic region may offer a physical explanation on the idea that type-*I* bifurcation in ref. [16] is possibly not suitable for cell differentiation. Interestingly, the EPR decreases monotonically along the change of parameter *S* from 0 to 1.5 (Fig. S3D). We speculated that this decrease of *S* may correspond to the carcinogenesis because that, according to the MEPR, the system will evolve from terminal expression states to a promiscuous expression state which is a well-known phenomenon in the carcinogenesis (Fig. S3B).

### Quantify the Dominant Developmental, Retrodifferentiation and Transdifferentional Paths on Two-gene Landscape

The center valley (A in Fig. 2a) on the verified development landscape corresponds to stem cells while the side valleys (B and C in Fig. 2a) correspond to two different terminal cell types. We quantified the dominant paths between valleys of different cell types. Similar to previous results [23], we first found that the development (paths from center valley A to marginal valleys B or C in Fig. 2a) and retrodifferentiation paths (paths from marginal valleys B or C to center valley A) over the landscape are distinct and irreversible. We then extended previous work [23] to quantify the dominant paths between two terminal cell types on the landscape. We assume them as biological transdifferentiation paths (paths between two marginal valleys B and C). By implementing the two searching methods introduced in the section of Material and Method, we reached a consistent dominant transdiferentiation path between two mature cell types (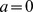), which indicates that the optimal approach to transdifferentiate needs to maintain the parameter staying at 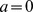 during the whole process. The transdifferentiation path is direct and just transverses from the starting valley on the landscape and directly moves towards the target valley without firstly moving towards the stem cell valley (Fig. 2). Moreover, the corresponding dominant paths between valleys B, and C over the developmental stage-specific landscape (parameter *a* is fixed to 0) are also nearly identical (Fig. 2b). These results drive us to speculate that the trandifferentiation may be reversible. However, at certain condition, we found that the transdifferentiation paths over developmental landscape will gradually move forward to the stem cells at first, then downward to the target cell type (data not shown). Applying these discoveries into the biological situation, it would imply that the optimal transdifferentiation strategy may depend on the source and target cell types as well as the the microenvironment where they locates in: some conversions need to firstly converse into the precursors then to the target cell types, while in extreme situation (here 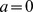 or no autocatalysis of gene expression) a direct phenotype conversion would be optimal.

We then varied the intensity of noise for whole dynamic landscape and found that the shape of landscape and the direction of dominant paths is apparently influenced by the intensity of gene expression noise (Fig. 3). When the noise of gene expression increases, the landscape becomes flatter and the barrier between different valleys becomes lower, thus the cell types become less stable. Accordingly, we found that the reprogramming and normal development paths become increasingly straight when the noise of the system increases from 0.1 to 1. Biologically, it indicates that the development, reprogramming processes could be accelerated when the gene expression stochasticity is magnified. In contrast to Waddington’s perspective about a static development landscape, this result could provide a dynamic view of the landscape [10],[12],[35].

Similar to previous studies, we further found that the mean first passage time (MFPT) for escaping from one valley decreases as noise increases [22] (Fig. S4), in line with the idea about the influence of the magnitude of developmental fluctuations due to the stochasticity of gene expression in ref. [18],[44],[45] (The equation of MFPT and the corresponding graph is included in the supporting information).

### Core CNS Developmental Network

Previous construction of development landscape only demonstrated on a general two-gene module which doesn’t consider multiple bifurcation of a specific biological system. In order to demonstrate the feasibility of the quantification of a landscape for a complex development process, we attempted to construct the developmental landscape for central nervous system development, starting from establishing a minimal gene regulatory network. In order to prove the validity of regulatory network as well as the model, we first implemented SDEs simulation and compared the simulated gene expression dynamics with microarray datasets. We also used the set of SDEs to simulate the transdifferentiation from oligodendrocyte/astrocyte to neurons and found that the simulation result matches conclusions drawn from the transdifferentiation paths of the two-gene landscape model. The two-step landscape was then constructed based on the confirmed SDEs of the coupled two fate switch circuits.

The minimal CNS differentiation network is shown in Fig. 4a. We notice that this network consists of the same two motifs (Fig. 4b), where there is a common upstream regulator positively regulating two mutual-inhibiting genes, a typical motif found in extensive network analysis in morphogenesis [46]. This motif is also used to explain the mechanism for stemness maintenance and reprogramming.

### Confirmation of the Minimal Network and Model for CNS Differentiation by Stochastic Simulation

We found that, when assigning 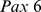 a high expression level and other genes low expression level at the beginning of the simulation, genes expressed sequentially in the network hierarchy in our stochastic simulation model, and captured the ordered developmental dynamics. Our result shows that the gene expression levels of 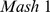 and 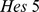 will first increase to a certain threshold, then follow a random switch between the expression of 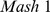 and 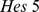 in the opposite direction which respectively commit to neuron or glia cellular fate, indicating a pitchfork-shape bifurcation in development (Fig. S5A). A similar bifurcation also appears for 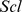 and 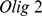 gene expression dynamics when the second step development occurs and cells commit to astrocyte or oligodendrocyte (Fig. S5A).

We found that our results qualitatively match the public microarray data. Taking single cell simulation of neuron differentiation as an example, the simulation agrees with public microarray data (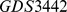) [41] in which the progression from undifferentiated neuron precursor at 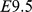 to neurogenesis at 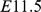 and to maturation of neurons at 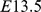 was measured (Fig. 5). In the microarray data, the expression of 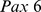 decreases constantly, while that of 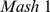 and 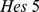 first increases from 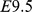 to 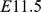. Then, 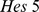 decreases dramatically from 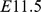 to 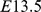 while 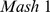 decreases only slightly in this time period. Our simulation shows the similar expression dynamics with a clear bifurcation between 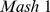 and 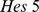 (Fig. S5A). Our simulation is further confirmed by the microarray data from 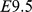 to 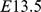, which shows that expression of markers of other cell types is much lower comparing to the expression of 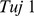. The stochastic simulation of the gene expression dynamics for astrogenesis and oligodendrogenesis also qualitatively agrees with the corresponding datasets of microarray experiments, as shown in the supporting information (Fig. S6–7).

We next explored the optimal “cocktails”of genes to be ecotypically expressed and their order under manipulation for converting astrocyte/oligodendrocyte to neuron [17]. By testing a serial of simulation, we found that the conversion could occur with higher efficiency when we activated sequentially 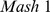, 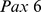, followed by inhibiting 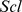 or 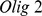 and 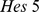. We further found that oligodendrocyte/astrocyte will firstly dedifferentiate into CNS precursor then redifferentiate into neurons during our transdifferentiation simulation (Fig. S5C). With strong enforced activation of 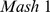, a direct transdifferentiations into neuron occurs in the simulation (data not shown). Our model also suggests that the cell types can automatically inter-converse between three cell types under large noise influence (data not shown). All the above three results agree with conclusion from the two-gene landscape model.

### Central Nervous System Developmental Landscape

Based on the above stochastic simulation confirmed model and network, we herein constructed the CNS developmental landscape. Of our knowledge, this is the first physical realization for constructing a two-step developmental landscape for a biological system.

Fig. 6b shows the first-step developmental landscape for 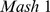 and 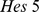 where the center valley corresponds to the CNS precursors, the left valley corresponds to the neuron cell type and the right valley corresponds to the glia cell type. Fig. 6c shows the second-step developmental landscape for 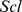 and 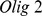 while the center valley corresponds to the glia, the left valley corresponds to the astrocyte and the right valley corresponds to the oligodendrocyte. Using an *ad hoc* coordinate system and gluing technique (see Material and method), we obtained the two-step development landscape by integrating the first and the second-step developmental landscapes (Fig. 6d). Our two-step CNS developmental landscape is similar to the original Waddington landscape (Fig. S1) with a two-step sequence of bifurcations that generates multiple valleys.

Under the same framework for the quantification of dominant paths over the two-gene landscape, we further explored the dominant paths for the development from the CNS progenitors to neuron/glia and from glia to astrocyte/oligodendrocyte, and the corresponding retrodifferentiation paths. Similar to the results of those from the two-gene landscape, the normal development paths on the CNS developmental lanscape are distinct from the retrodifferentiation paths. In addition, by using the searching method applied in the two-gene network (the 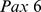 corresponds to the parameter *a* in two-gene landscape), we found the dominant transdifferentiation paths on the landscape need firstly move back to the CNS progenitor then downward the target terminal cell types (data not shown). These discoveries are similar to the results from the stochastic simulation where the reprogramming cocktails of genes will firstly reprogram the source cell types into CNS progenitors then into the target cell types.

## Discussion

The studies on the two-gene landscape provide a few interesting insights into the mechanism of development, retrodifferentiation as well as transdifferentiation.

We demonstrated that the development and retrodifferentiation paths are irreversible because the dominant paths don’t follow gradient since there is an additional curl force determining the dynamics of the system [23]. Such an irreversibility of forward and backward paths may be a common phenomenon in the non-equilibrium systems, like the living organisms. This conclusion can be extended to embrace recent findings that the genomic gene expression dynamics during developmental fibroblast formation is distinct from that in the iPSCs induction [47].

Intriguingly, a direct transdifferentiation path without passing through the medial precursor states appears on the landscape at certain development stage (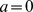). Meanwhile, in certain range of parameter *a*, the transdifferentiation path will firstly move toward the stem cell valley and then to the target cell valley. These results may well associate with previous ideas (as also shown in our transdifferentiation simulation) that the transdifferentiation can happen through firstly dedifferentiating into precursors, then redifferentiating into the target cells. At extreme situation, a direct phenotypic conversion is applicable. It can be related to recent findings that, under additional signal induction, the direct conversion between fibroblast and neural progenitor has a higher efficiency than that by first reprogramming to the stem cells, followed by instructing to target cell types [48].

Our work demonstrates the importance of gene-expression stochasticity in development. With higher noise term in the SDEs system, the cell types can automatically inter-converse (data not shown); similarly, a larger diffusion matrix **D** will flatten the landscape, and lead to straight dominant developmental/retrodifferentiational paths and convenient transition between different valleys (cell types) over developmental landscape. These results reflect previous considerations that the reprogramming efficiency may be improved when biological noise processing mechanism (Proteasome or Wnt signal pathway, *et al.*) is enhanced [49].

In practise of iPSCs, the somatic cells are induced by adding ecto-expressing factors (OKSM, *Oct 4, Klf 4, Sox 8, Myc*). Previous studies [23] for explaining the reprogramming by two-gene model (Fig. 1a) with a parallel increase of parameter 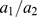 in Eq. 1 didn’t explicitly consider the ecto-expression of pluripotent genes. In that explanation all the two fate genes will finally return to low expression level. The enforced expression of OSKM in reprogramming cells may be safely explained by an improved perspective. The mutually-inhibited genes in the two-gene model are possibly a part of the complex differentiation network responsible for different somatic cell types fate commitment. They are further activated by the pluripotency network genes including the OKSM. A simplified model for this idea is shown in Fig. 4b as a three-gene model. In this model, we found that, when we enforce the expression of upstream regulator, the side terminal cells can be indeed reprogrammed into states of center stem cells (Fig. S8).

Our work is different from that Wang’s in considerable ways. We extended Wang’s study to construct the 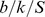-change landscape and provided physical explanation why 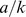 change can be used to map a developmental process while 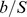 change cannot. We also quantified the transdifferentiation paths over the two-gene landscape and found they are identical and direct (parameter *a* doesn’t change). In contrast to Wang’s one-step landscape, we also constructed the first two-step Waddington development landscape based on a manual-curated network of CNS development.

There are several relevance and differences between our CNS developmental landscape and the original Waddington landscape. Similar to the original landscape, our CNS developmental landscape has two-step bifurcation with multiple valleys. Each valley corresponds to a specific CNS cell type (Fig. 6d). Like what shown on the Waddington landscape, the development on CNS landscape starts from the center valley and ends with the formation of three CNS cell type after two-step bifurcation. However, developmental landscape in Waddingtonnian sense is static [10] while our landscape is dynamic due to the noise influence. The dominant transdifferentiation paths over CNS developmental landscape do not correspond to a direct phenotypic switch as in the two-gene landscape. This may result from the fact that the bifurcation on the CNS developmental landscape increases from one stable steady states to two stable states (Fig. 6a) while that of the two-gene developmental landscape decreases from three stables states to two stable steady states (Fig. 1b, c).

Previous studies model the irreversibility of development by certain feedback loops [49],[50]. Here we assumed an exponential decrease of 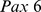 to represent the feedback from all the mature cell types. But we also considered supposed direct feedback from marker genes (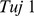, 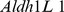 and 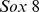) to 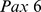 and obtained similar results. In hematopoiesis, the gene expression dynamics of multiple-step two-gene motifs is investigated by stochastic simulation and bifurcation analysis in ref. [49],[51]. However, we herein present a physical model for CNS development, thus provide a much fundamental and global understanding of cellular development which cannot be captured by stochastic simulation and bifurcation analysis.

## Supporting Information

Figure S1 **The original developmental/epigenetic landscape proposed by Cornad Waddington** [**10**] **.** The original epigenetic landscape. Here the landscape concept refers to the recurring metaphor describing differentiation as a “down-hill” process of a cell “rolling” down from the high pluripotent hilltop (the embryonic stem cells) to the lower valleys (the terminal differentiated cells).
(EPS)

Figure S2 **Landscape constructed by Ao or Bhattacharya’s method.** The stage-specific landscapes constructed by Ao or Bhattacharya’s methods are topologically similar to what presented in the main text. However, only the development landscape constructed by Ao’s method is similar to what shown in the main text. (A, C) Stage-specific (parameter *a* fixed) two-gene landscape quantified by Bhattacharya and Ao’s methods (B, D) The developmental landscape by Bhattacharya and Ao’s method.
(EPS)

Figure S3
**The two-gene whole dynamic landscape and EPR evolution under** ***b*** **or** ***S*** **change** **TOP.** Similar landscapes are constructed by systematically changing parameters b/S with other parameters fixed (A) b-change landscape (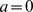) (B) S-change landscape **Bottom**: Under the change of parameter *b*, EPR doesn’t increase monotonically in the starting part, providing a potential physical explanation about why type-I bifurcation ([16]) is not suitable for development. EPR decreases monotonically along the increase of parameter *S*, which implies that the decrease of parameter *S* may correspond to the carcinogenesis (See main text). (C) EPR vs *b* increases from 0 to 1.5. (D) EPR vs *S* increases from 0.3 to 1. Here we also applied the basic parameters settings as in Figure 1 of main text. EPR: Entropy Production Rate(EPS)

Figure S4 **The mean first passage time (MFPT or** *t* **) from side attractor to center attractor under different noises.** As the noise increases, the developmental landscape becomes increasingly flat (see Figure 3 in maintext). Accordingly, MFPT decreases, which implies the cell types become less stable.
(EPS)

Figure S5
**Stochastic simulation of CNS differentiation by two different model and reprogramming from oligodendrocyte to neuron.** (A) The gene expression dynamics during CNS differentiation which is epitomized by two step bifurcation, similar to that of pancreas development as shown by previous studies [17]. This diagram is based on our default model with an enforced decrease of 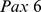. (B) The same as subfigure A, but based on an alternative model with the negative feedback from marker genes to CNS progenitor gene 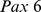. (C) The gene expression dynamics during reprogramming from oligodendrocyte to neuron. It shows that the oligodendrocyte first needs to be induced into precursors during the reprogramming. The similar results applies for reprogramming astrocyte to neuron.(EPS)

Figure S6 **Results of stochastic simulation matches qualitatively public microarray datasets for astrogenesis.** The microarray datasets confirmed qualitatively the starting part of the simulation for astrogenesis gene expression dynamics. (A) Microarray data for astrogenesis from E11 to E14. Each box represents a specific time period, in which there are two duplications of samples. Diagrams in row a and b shows the gene expression dynamics for glia inducer and the second-step switch genes, and astrogenesis related regulators/markers, respectively. The expression level of each gene is normalized by the corresponding maxima in the time series. (B) Relative expression dynamics of three cell types markers in astrogenesis by microarray data. (C) Single cell simulation for astrogenesis where the starting part of the diagrams in column a and b matches quantitatively with the gene expression dynamics of the diagrams in the according rows of subgraph A.
(EPS)

Figure S7 **Results of Stochastic simulation matches qualitatively public microarray datasets for oligodendrogenesis.** The microarray datasets confirmed qualitatively the end part of the simulation for oligodendrogenesis gene expression dynamics. (A) Microarray data for oligodendrogenesis from E11 to E14. Each box represents a specific time period, in which there are five duplications of samples. This diagram shows the gene expression dynamics for second-step differentiation and oligodencrogenesis related regulators/markers, respectively. The expression level of each gene is normalized by the corresponding maxima in the time series. (B) Relative expression dynamics of three cell types markers in oligodendrogenesis by microarray data. (C) Single cell simulation for oligodendrogenesis where the late part of the simulation matches quantitatively with the gene expression dynamics shown subgraph a.
(EPS)

Figure S8 **The bifurcation and developmental landscape of three-gene model.** The bifurcation graph is based on the three-gene motif of Figure 4b in main text, used to explain our proposed idea about the stem cell stemness maintenance. The developmental landscape is calculated through systematically changing the expression level of gene A in the three-gene motif. To obtain the figures, equation S7–8 is applied. (A) The bifurcation graph of the three-gene motif where the dark points are the stable fixed points and the light points are the unstable fixed points. (B) The developmental landscape of the three-gene motif.
(EPS)

Table S1 **Crucial genes and regulatory relationship for CNS development by manually curation.** In this table, the *Source regulator* column shows the upstream genes and the *Target gene* shows their corresponding targets, both of which are crucial genes underling CNS development. The relationship between regulators and targets is listed in the third column, either positive (up-regulation) or negative (down-regulation). If the regulation is proposed, we denote a “FALSE” item in the fourth column. For the confirmed regulation, we list the reference (Pubmid) for readers’ check, while for proposed regulation, the listed reference evidenced the importance of the target genes in the formation of CNS. The data in this table is used to draw the Figure 4a in main text.
(PDF)

Supporting information S1 **This combined fourteen pages supporting file includes four sections about the mathematical basis for Ao’s and Sbhattacharya’s approaches to landscape construction, calculation of MFPT under different noise influence, the confirmation of astrogenesis and oligodendrogenesis simulations by comparing with microarray data and the proposal for explaining the stemness maintenance by the three-gene model, respectively.**
(PDF)

## References

[1] Takahashi K, Yamanaka S (2006) Induction of pluripotent stem cells from mouse embryonic and adult fibroblast cultures by defined factors. Cell 126: 663–676. 16904174 10.1016/j.cell.2006.07.024

[2] Wernig M, Meissner A, Foreman R, Brambrink T, Ku M, et al (2007) In vitro reprogramming of fibroblasts into a pluripotent es-cell-like state. NATURE-LONDON- 448: 318. 17554336 10.1038/nature05944

[3] Liao J, Wu Z, Wang Y, Cheng L, Cui C, et al (2008) Enhanced efficiency of generating induced pluripotent stem (ips) cells from human somatic cells by a combination of six transcription factors. Cell research 18: 600–603. 18414447 10.1038/cr.2008.51

[4] Davis R, Weintraub H, Lassar A (1987) Expression of a single transfected cdna converts fibroblasts to myoblasts. Cell 51: 987–1000. 3690668 10.1016/0092-8674(87)90585-x

[5] Vierbuchen T, Ostermeier A, Pang Z, Kokubu Y, Südhof T, et al (2010) Direct conversion of fibroblasts to functional neurons by defined factors. Nature 463: 1035–1041. 20107439 10.1038/nature08797 PMC2829121

[6] Xie H, Ye M, Feng R, Graf T (2004) Stepwise reprogramming of b cells into macrophages. Cell 117: 663–676. 15163413 10.1016/s0092-8674(04)00419-2

[7] González F, Boué S, Belmonte J (2011) Methods for making induced pluripotent stem cells: reprogramming a la carte. Nature Reviews Genetics 12: 231–242. 10.1038/nrg2937 21339765

[8] Ruan W, Han J, Li P, Cao S, An Y, et al (2011) A novel strategy to derive ips cells from porcine fibroblasts. SCIENCE CHINA Life Sciences 54: 553–559. 21706416 10.1007/s11427-011-4179-5

[9] Huang S (2009) Reprogramming cell fates: reconciling rarity with robustness. Bioessays 31: 546–560. 19319911 10.1002/bies.200800189

[10] Waddington C, Kacser H (1957) The strategy of the genes. Allen and Unwin.

[11] Jablonka E, Lamb M (2002) The changing concept of epigenetics. Annals of the New York Academy of Sciences 981: 82–96. 12547675 10.1111/j.1749-6632.2002.tb04913.x

[12] Huang S (2011) On the intrinsic inevitability of cancer: From foetal to fatal attraction. In: Seminars in cancer biology. 10.1016/j.semcancer.2011.05.003 21640825

[13] Thom R (1954) Quelques propriétés globales des variétés différentiables. Commentarii Mathematici Helvetici 28: 17–86.

[14] Kauffman S (1969) Homeostasis and differentiation in random genetic control networks. Nature 224: 177–178. 5343519 10.1038/224177a0

[15] Kauffman S (1969) Metabolic stability and epigenesis in randomly constructed genetic nets. Journal of theoretical biology 22: 437–467. 5803332 10.1016/0022-5193(69)90015-0

[16] Huang S, Guo Y, May G, Enver T (2007) Bifurcation dynamics in lineage-commitment in bipotent progenitor cells. Developmental biology 305: 695–713. 17412320 10.1016/j.ydbio.2007.02.036

[17] Zhou J, Brusch L, Huang S (2011) Predicting pancreas cell fate decisions and reprogramming with a hierarchical multi-attractor model. PloS one 6: e14752. 21423725 10.1371/journal.pone.0014752 PMC3056652

[18] Chang H, Hemberg M, Barahona M, Ingber D, Huang S (2008) Transcriptome-wide noise controls lineage choice in mammalian progenitor cells. Nature 453: 544–547. 18497826 10.1038/nature06965 PMC5546414

[19] Wang J, Xu L, Wang E, Huang S (2010) The potential landscape of genetic circuits imposes the arrow of time in stem cell differentiation. Biophysical journal 99: 29–39. 20655830 10.1016/j.bpj.2010.03.058 PMC2895388

[20] Ao P (2009) Global view of bionetwork dynamics: adaptive landscape. Journal of Genetics and Genomics 36: 63–73. 19232305 10.1016/S1673-8527(08)60093-4 PMC3165055

[21] Ao P (2005) Laws in darwinian evolutionary theory. Physics of life Reviews 2: 117–156.

[22] Wang J, Xu L, Wang E (2008) Potential landscape and ux framework of nonequilibrium networks: robustness, dissipation, and coherence of biochemical oscillations. Proceedings of the National Academy of Sciences 105: 12271. 10.1073/pnas.0800579105 PMC2527901 18719111

[23] Wang J, Zhang K, Xu L, Wang E (2011) Quantifying the waddington landscape and biological paths for development and differentiation. Proceedings of the National Academy of Sciences 108: 8257. 10.1073/pnas.1017017108 PMC3100956 21536909

[24] Bhattacharya S, Zhang Q, Andersen M (2011) A deterministic map of waddington’s epigenetic landscape for cell fate specification. BMC Systems Biology 5: 85. 21619617 10.1186/1752-0509-5-85 PMC3213676

[25] Xing J (2010) Mapping between dissipative and hamiltonian systems. Journal of Physics A: Mathematical and Theoretical 43: 375003.

[26] Ao P (2004) Potential in stochastic differential equations: novel construction. Journal of physics A: mathematical and general 37: L25.

[27] Kwon C, Ao P, Thouless D (2005) Structure of stochastic dynamics near fixed points. Proceedings of the National Academy of Sciences of the United States of America 102: 13029. 16141337 10.1073/pnas.0506347102 PMC1201617

[28] Ao P, Kwon C, Qian H (2007) On the existence of potential landscape in the evolution of complex systems. Complexity 12: 19–27.

[29] Yin L, Ao P (2006) Existence and construction of dynamical potential in nonequilibrium processes without detailed balance. Journal of Physics A: Mathematical and General 39: 8593.

[30] Qiang M, Wu B, Liu Y (2011) A brief review on current progress in neuroscience in china. SCIENCE CHINA Life Sciences 54: 1156–1159. 22227910 10.1007/s11427-011-4261-z

[31] Van Ooyen A (2003) Modeling neural development. The MIT Press.

[32] Graf T, Enver T (2009) Forcing cells to change lineages. Nature 462: 587–594. 19956253 10.1038/nature08533

[33] Zhou J, Huang S (2010) Understanding gene circuits at cell-fate branch points for rational cell reprogramming. Trends in Genetics. 10.1016/j.tig.2010.11.002 21146896

[34] Andrecut M, Halley J, Winkler D, Huang S (2011) A general model for binary cell fate decision gene circuits with degeneracy: Indeterminacy and switch behavior in the absence of cooperativity. PloS one 6: e19358. 21625586 10.1371/journal.pone.0019358 PMC3098230

[35] Huang S (2011) The molecular and mathematical basis of waddington’s epigenetic landscape: A framework for post-darwinian biology? BioEssays. 10.1002/bies.201100031 22102361

[36] Swenson R, Turvey M (1991) Thermodynamic reasons for perception–action cycles. Ecological Psychology 3: 317–348.

[37] Wang J, Xu L, Wang E (2008) Robustness, dissipations and coherence of the oscillation of circadian clock: potential landscape and flux perspectives. PMC biophysics 1: 7. 19351381 10.1186/1757-5036-1-7 PMC2667439

[38] Wang J, Zhang K, Wang E (2010) Kinetic paths, time scale, and underlying landscapes: A path integral framework to study global natures of nonequilibrium systems and networks. The Journal of chemical physics 133: 125103. 20886967 10.1063/1.3478547

[39] Bertrand N, Castro D, Guillemot F (2002) Proneural genes and the specification of neural cell types. Nature Reviews Neuroscience 3: 517–530. 12094208 10.1038/nrn874

[40] Sayers E, Barrett T, Benson D, Bolton E, Bryant S, et al (2011) Database resources of the national center for biotechnology information. Nucleic acids research 39: D38–D51. 21097890 10.1093/nar/gkq1172 PMC3013733

[41] Hartl D, Irmler M, Römer I, Mader M, Mao L, et al (2008) Transcriptome and proteome analysis of early embryonic mouse brain development. Proteomics 8: 1257–1265. 18283662 10.1002/pmic.200700724

[42] Sanosaka T, Namihira M, Asano H, Kohyama J, Aisaki K, et al (2008) Identification of genes that restrict astrocyte differentiation of midgestational neural precursor cells. Neuroscience 155: 780–788. 18640244 10.1016/j.neuroscience.2008.06.039

[43] Nielsen J, Maric D, Lau P, Barker J, Hudson L (2006) Identification of a novel oligodendrocyte cell adhesion protein using gene expression profiling. The Journal of neuroscience 26: 9881. 17005852 10.1523/JNEUROSCI.2246-06.2006 PMC1613258

[44] Brock A, Chang H, Huang S (2009) Non-genetic heterogeneitya mutation-independent driving force for the somatic evolution of tumours. Nature Reviews Genetics 10: 336–342. 10.1038/nrg2556 19337290

[45] Arias A, Hayward P (2006) Filtering transcriptional noise during development: concepts and mechanisms. Nature Reviews Genetics 7: 34–44. 10.1038/nrg1750 16369570

[46] Cotterell J, Sharpe J (2010) An atlas of gene regulatory networks reveals multiple three-gene mechanisms for interpreting morphogen gradients. Molecular systems biology 6. 10.1038/msb.2010.74 PMC3010108 21045819

[47] Jopling C, Boue S, Belmonte J (2011) Dedifferentiation, transdifferentiation and reprogramming: three routes to regeneration. Nature Reviews Molecular Cell Biology 12: 79–89. 21252997 10.1038/nrm3043

[48] Kim J, Efe J, Zhu S, Talantova M, Yuan X, et al (2011) Direct reprogramming of mouse fibroblasts to neural progenitors. Proceedings of the National Academy of Sciences 108: 7838. 10.1073/pnas.1103113108 PMC3093517 21521790

[49] MacArthur B, Please C, Oreffo R (2008) Stochasticity and the molecular mechanisms of induced pluripotency. PLoS One 3: e3086. 18769478 10.1371/journal.pone.0003086 PMC2517845

[50] Chickarmane V, Enver T, Peterson C (2009) Computational modeling of the hematopoietic erythroid-myeloid switch reveals insights into cooperativity, priming, and irreversibility. PLoS computational biology 5: e1000268. 19165316 10.1371/journal.pcbi.1000268 PMC2613533

[51] Krumsiek J, Marr C, Schroeder T, Theis F (2011) Hierarchical differentiation of myeloid progenitors is encoded in the transcription factor network. PloS one 6: e22649. 21853041 10.1371/journal.pone.0022649 PMC3154193

