# Supplementary material for: From Understanding the Development Landscape of the Canonical Fate-Switch Pair to Constructing a Dynamic Landscape for Two-Step Neural Differentiation
Source: PLoS One. 2012 Dec 4;7(12):e49271. doi: 10.1371/journal.pone.0049271 (PMC3530918; doi:10.1371/journal.pone.0049271)
Supplement: Table S1 — Crucial genes and regulatory relationship for CNS development by manually curation. In this table, the Source regulator column shows the upstream genes and the Target gene shows their corresponding targets, both of which are crucial genes underling CNS development. The relationship between regulators and targets is listed in the third column, either positive (up-regulation) or negative (down-regulation). If the regulation is proposed, we denote a “FALSE” item in the fourth column. For the confirmed regulation, we list the reference (Pubmid) for readers’ check, while for proposed regulation, the listed reference evidenced the importance of the target genes in the formation of CNS. The data in this table is used to draw the Figure 4a in main text. (PDF) [file pone.0049271.s009.pdf]

Source regulator	*Target gene*	*Regulation type*	*Experimental confirmation*	*Evidences (Pubmid)*
Pax6	Hes5	up	TRUE	19521200
Pax6	Mash1	up	TRUE	17344230
Mash1	Hes5	down	TRUE	10952889; 17006542
Hes5	Mash1	down	TRUE	18344989;17409112; 11331769;10804175
Mash1	Zic1	up	TRUE	17501568
Mash1	Brn2	up	FALSE	21185279
Zic1	Tuj1	up	FALSE	18171944
Brn2	Tuj1	up	FALSE	18171944
Myt1L	Tuj1	up	TRUE	15935060
Hes5	Olig2	up	TRUE	18816798; 18287207
Hes5	Scl	up	TRUE	20672298; 20368359; 15156153
Hes5	Stat3	up	TRUE	15156153
Olig2	Scl	down	TRUE	21068830
Scl	Olig2	down	TRUE	21068830
Brn2	Olig2	down	FALSE	21068830
Olig2	Brn2	down	FALSE	21185279
Scl	Stat3	up	TRUE	21633967
Stat3	AldhlL1	up	TRUE	18171944
Olig2	Myt1L	up	TRUE	9373037
Olig2	Sox8	up	TRUE	15102707
