# Supplementary material for: From Understanding the Development Landscape of the Canonical Fate-Switch Pair to Constructing a Dynamic Landscape for Two-Step Neural Differentiation
Source: PLoS One. 2012 Dec 4;7(12):e49271. doi: 10.1371/journal.pone.0049271 (PMC3530918; doi:10.1371/journal.pone.0049271)
Supplement: Supporting information S1 — This combined fourteen pages supporting file includes four sections about the mathematical basis for Ao’s and Sbhattacharya’s approaches to landscape construction, calculation of MFPT under different noise influence, the confirmation of astrogenesis and oligodendrogenesis simulations by comparing with microarray data and the proposal for explaining the stemness maintenance by the three-gene model, respectively. (PDF) [file pone.0049271.s010.pdf]

From understanding the development landscape of the canonical fate-switch pair to constructing a dynamic landscape for two-step neural differentiation

Supporting Information

Xiaojie Qiu[1,$], Shanshan Ding[1,$], Tieliu Shi[1,*],

October 25, 2012

1. Center for Bioinformatics and Computational Biology, and The Institute of Biomedical Sciences, School of Life Sciences, East China Normal University, 500 Dongchuan Road, Shanghai 200241, China.

$ These authors contributed equally to this work

Contact: Tuexy.xiaojie@gmail.com, dsskaixuan@gmail.com or tieliushi01@gmail.com

Two-gene developmental landscape: the two remaining constructions

A caveat of Wang's construction is that the extreme points on landscape deviate from the locality of equilibrium points of equation (1) in main text because the Itô stochastic integral is used in his construction. Ao proposed a framework based on symmetric and antisymmetric matrix transformation to decompose the SDEs into the gradient part and curl part, which ensures the extreme points on landscape match the stable points (Ao, 2003). However, a SDEs decomposition by this transformation is not easy even though Ao developed a gradient expansion algorithm (Ao, 2004). There also existed another effective method for landscape construction (Bhattacharya *et al*, 2011). But it is only a numerical method and doesn't consider the influence of noise. Considering the above situations, we mainly applied Wang's method of landscape construction. Here we summarize the main mathematical basis for landscape construction by Ao and Bhattacharya's methods.

For Ao's transformation, let us first consider a process defined by following SDEs (Xing, 2010):

$$\mathrm{d}x_i/\mathrm{d}t \quad = \quad G_i(\mathbf{x}) + \epsilon \sum_{j=1}^{m} g_{ij}(\mathbf{x})\varsigma_j(t), i = 1, ..., n. \qquad \text{(S1)}$$

where g relates to diffusion matrix $\mathbf{D}$ by $\mathbf{g}\mathbf{g}^{\mathrm{T}} = 2\mathbf{D}$, the independent Gaussian white noise satisfies $< \varsigma_j(t), \varsigma_j(\tau) >= \delta_{ij}\delta(t-\tau)$ and ϵ is used to characterize the intensity of noise. Here G_i corresponds to the F_i (the force driving gene i) in the main text while $\mathrm{d}x_i/\mathrm{d}t$ describes the change of rate in gene expression of gene i. In order to obtain a scalar function corresponding to the potential function for the SDEs system, one can always construct a symmetric matrix $\mathbf{S}$ and antisymmetric matrix $\mathbf{T}$ and transform Eq. S1 into:

$$\begin{aligned}(\mathbf{S}+\mathbf{T}) \cdot \frac{d\mathbf{x}}{dt} &= (\mathbf{S}+\mathbf{T}) \cdot (\mathbf{G}(\mathbf{x}) + \epsilon\mathbf{g}(\mathbf{x}) \cdot \varsigma(t))) \\ &= -\nabla_{\mathbf{x}}\phi(\mathbf{x}) + \epsilon\mathbf{g}^{'}(\mathbf{x}) \cdot \varsigma(t) \qquad \text{(S2)}\end{aligned}$$

where ϕ is the scalar potential function. By introducing an additional condition, $\mathbf{g}^{'}\mathbf{g}^{'\mathrm{T}} = 2\mathbf{S}$, we can uniquely determine the matrixes of $\mathbf{S}$ and $\mathbf{T}$ by

$$\partial \times [(\mathbf{M^{-1}} \cdot \mathbf{G}(\mathbf{x}))] = 0, \mathbf{M} + \mathbf{M}^{\mathrm{T}} = 2\mathbf{D} \qquad \text{(S3)}$$

, where $\mathbf{M^{-1}} = \mathbf{S} + \mathbf{T}$.

To obtain $\mathbf{M}$, a gradient expansion algorithm is developed:

$$\mathbf{M} = \mathbf{D} + \Delta \lim_{j\to\infty} M_j \qquad \text{(S4)}$$

with $\Delta M_j = \sum_{l=1}^{\infty}(-1)^l[(F^{\tau})^l\tilde{D}_jF^{-l} + (F^{\tau})^{-l}\tilde{D}_jF^l], \tilde{D}_0 = \mathbf{D}F - F^{\tau}\mathbf{D}, \tilde{D}_{j\geq 1} = (\mathbf{D} + \Delta M_{j-1})\{[\partial \times (\mathbf{D}^{-1} + \Delta M_{j-1}^{-1})]\mathbf{G}\}(\mathbf{D} - \Delta M_{j-1})$. The zeroth order approximation to equation S3 is $\mathbf{M}\mathbf{F}^{\mathrm{T}} - \mathbf{F}\mathbf{M}^{\mathrm{T}} = 0$ (Ao, 2004), where $F_{ij} = \frac{\partial G_i}{\partial x_j}, i, j = 1...n$. Based on the solution of $\phi(\mathbf{x})$, the landscape for the system can be mapped.

According to Bhattacharya's method of landscape construction (Bhattacharya *et al*, 2011), for a two genes system described by Eq. 1 in main text, we can define a quasi-potential term V_q whose increment is

$$\begin{aligned}\Delta V_q &= \frac{\partial V_q}{\partial x_1} \cdot \Delta x_1 + \frac{\partial V_q}{\partial x_2} \cdot \Delta x_2 \\ &= -\frac{\mathrm{d}x_1}{\mathrm{d}t}\Delta x_1 - \frac{\mathrm{d}x_2}{\mathrm{d}t}\Delta x_2 \\ &= -\frac{\mathrm{d}x_1}{\mathrm{d}t}\Big(\frac{\mathrm{d}x_1}{\mathrm{d}t}\Delta t\Big) - \frac{\mathrm{d}x_2}{\mathrm{d}t}\Big(\frac{\mathrm{d}x_2}{\mathrm{d}t}\Delta t\Big) \\ &= -\Big[\Big(\frac{\mathrm{d}x_1}{\mathrm{d}t}\Big)^2 + \Big(\frac{\mathrm{d}x_2}{dt}\Big)^2\Big]\Delta t \qquad \text{(S5)}\end{aligned}$$

By aligning all the trajectories under the assumption of the continuity of potential, we can then map the landscape for the above system.

We first constructed the stage-specific and developmental landscape by Ao's framework and Bhattacharya's method (Figure S2) with the same treatment of parameter a in Wang's framework (Wang *et al*,

2011). We found that the stage-specific landscapes constructed are almost topologically identical. This may result from the fact that both constructions tightly associate with the theory of Lyapunov stability (Bhattacharya *et al*, 2011, Yuan *et al*, 2010). By similar operation stated in the main text, we further quantified the development landscape. We found that the quasi-potential on Bhattacharya's developmental landscape increases from stem cells to mature cell types which is contrary to the original Waddington landscape. On the other hand, the quasi-potential on Ao's developmental landscape firstly decreases from the stem cell valley then increases slightly.

Mean first passage time under different noise influence

To characterize the stability of cell types under different noise amplitude, we use the concept of mean first passage time (MFPT) which is defined as (van Kampen, 2007)

$$F_1 \frac{\partial \tau}{\partial x_1} + F_2 \frac{\partial \tau}{\partial x_2} + D\left(\frac{\partial^2 \tau}{\partial x_1^2} + \frac{\partial^2 \tau}{\partial x_2^2}\right) = -1 \quad (\text{S6})$$

, where F_1, F_2, x_1, x_2 is the same terms as in the equation (1) from the main text. τ is the the mean first passage time for a cell stays at a specific valley (corresponds to a cell type) on landscape (Wang *et al*, 2010). From Figure S4, we find that the MFPT decreases as the noise amplitude increases, implying that the cell types become less stable under large noise influence.

Confirmation of stochastic simulation by public microarray data

We used two different models (the first one has an enforced decrease of Pax 6 and another has the negative feedback from mature cell type to Pax 6) to simulate the development dynamics of CNS genesis and obtained similar results (Figure S5A, B). We used the first one as our default model.

In addition to a qualitative consistency between the stochastic simulation of neurongenesis and the microarray data, as shown in the main text, the stochastic simulation for astrogenesis and oligodendrogenesis also partially matches qualitatively with the public microarray time series.

The expression of Scl, $Olig$ 2 and Hes 5 increases from $E11$ to $E14$ in the microarray data ($GDS10796$) for astrogenesis (Figure S6). This may correspond to the starting part of our simulation. The expression of $Stat$ 3, $Aldh1L1$ and Sox 8 increases simultaneously in the microarray data, identifying with the temporal increase both of Scl and $Olig$ 2 (before the bifurcation of their expression) caused by Hes 5 activation in the starting part of the simulation. It also indicates that the astrogenesis may begin at about $E11$ (Figure S6). The expression of $Aldh1L$ is relative low, comparing to Tuj 1 and Sox 8, which may result from the fact that $Aldh1L$ is not the optimal marker for astrocytes (Cotterell and Sharpe, 2010).

As confirmed by the microarray data ($GDS2379$), the expression level of Scl increases while that of $Olig$ 2 decreases during oligodendrogenesis in the later part of our simulation (Figure S7). The decrease of $MytlL$ and Sox 8 is at odds with our simulation, possibly because that they are also not the optimal markers of oligodendrocytes.

We also conducted reprogramming simulation after confirming our model and network by the above stochastic simulation (Figure. S5C).

Three-gene motif as the network design for stemness maintenance

In the main text, we proposed that the three-gene motif in Figure 4b is the network design for stemness maintenance and cellular development. To model this motif, we consider to couple the upstream gene's positive regulation and self-activation and using following equations to define its expression dynamics:

$$\frac{dx_1}{dt} = a_1 \frac{x_1^n}{S_1^n + x_1^n} \cdot \frac{x_3^n}{S_2^n + x_3^n} + \frac{b_1 S_1^n}{S_1^n + x_2^n} - k_1 x_1 = F_1(x_1, x_2) \quad (\text{S7})$$

$$\frac{dx_2}{dt} = a_2 \frac{x_2^n}{S_1^n + x_2^n} \cdot \frac{x_3^n}{S_2^n + x_3^n} + \frac{b_2 S_1^n}{S_1^n + x_1^n} - k_2 x_2 = F_2(x_1, x_2) \quad (\text{S8})$$

, where x_1, x_2, x_3 represent the gene expression level of b, c, a of the three-gene motif in Figure 4b of main text. We set $a_1 = a_2 = 1, b_1 = b_2 = 1, k_1 = k_2 = 1,\ S_1 = 0.5, S_2 = 2, n = 4$ for this three-gene model. Figure S8 is bifurcation graph and the corresponding development landscape calculated based on equations of x_1, x_2 under the change of x_3 (assumed to represent the developmental process). From this figure, we can conclude that the two side terminal cell type valleys can be reprogrammed to center stem cell valley by the enforced increase of gene A.

References

Ao P (2003) Stochastic force defined evolution in dynamical systems. *Arxiv preprint physics0302081*

Ao P (2004) Potential in stochastic differential equations: novel construction. *Journal of physics A mathematical and general* **37**: L25

Bhattacharya S, Zhang Q, Andersen M (2011) A deterministic map of Waddington's epigenetic landscape for cell fate specification. *BMC Systems Biology* **5**: 85

Cotterell J, Sharpe J (2010) An atlas of gene regulatory networks reveals multiple three-gene mechanisms for interpreting morphogen gradients. *Molecular systems biology* **6**

van Kampen N (2007) *Stochastic processes in physics and chemistry*. North holland

Wang J, Xu L, Wang E, Huang S (2010) The potential landscape of genetic circuits imposes the arrow of time in stem cell differentiation. *Biophysical journal* **99**: 29–39

Wang J, Zhang K, Xu L, Wang E (2011) Quantifying the Waddington landscape and biological paths for development and differentiation. *Proceedings of the National Academy of Sciences* **108**: 8257

Xing J (2010) Mapping between dissipative and Hamiltonian systems. *Journal of Physics A Mathematical and Theoretical* **43**: 375003

Yuan R, Ma Y, Yuan B, Ao P (2010) Constructive Proof of Global Lyapunov Function as Potential Function. *Arxiv preprint arXiv10122721*
